# Heme Signaling Impacts Global Gene Expression, Immunity and Dengue Virus Infectivity in *Aedes aegypti*

**DOI:** 10.1371/journal.pone.0135985

**Published:** 2015-08-14

**Authors:** Vanessa Bottino-Rojas, Octávio A. C. Talyuli, Natapong Jupatanakul, Shuzhen Sim, George Dimopoulos, Thiago M. Venancio, Ana C. Bahia, Marcos H. Sorgine, Pedro L. Oliveira, Gabriela O. Paiva-Silva

**Affiliations:** 1 Instituto de Bioquímica Médica Leopoldo de Meis, Universidade Federal do Rio de Janeiro, Rio de Janeiro, 21941–902, Brazil; 2 Instituto Nacional de Ciência e Tecnologia em Entomologia Molecular (INCT-EM), Rio de Janeiro, Brazil; 3 W. Harry Feinstone Department of Molecular Microbiology and Immunology, Bloomberg School of Public Health, Johns Hopkins University, Baltimore, MD, 21205–2179, United States of America; 4 Genome Institute of Singapore, Singapore,138672, Singapore; 5 Centro de Biociências e Biotecnologia, Universidade Estadual do Norte Fluminense Darcy Ribeiro, Campos dos Goytacazes, 28013–602, Brazil; CINVESTAV-IPN, MEXICO

## Abstract

Blood-feeding mosquitoes are exposed to high levels of heme, the product of hemoglobin degradation. Heme is a pro-oxidant that influences a variety of cellular processes. We performed a global analysis of heme-regulated *Aedes aegypti* (yellow fever mosquito) transcriptional changes to better understand influence on mosquito physiology at the molecular level. We observed an iron- and reactive oxygen species (ROS)-independent signaling induced by heme that comprised genes related to redox metabolism. By modulating the abundance of these transcripts, heme possibly acts as a danger signaling molecule. Furthermore, heme triggered critical changes in the expression of energy metabolism and immune response genes, altering the susceptibility towards bacteria and dengue virus. These findings seem to have implications on the adaptation of mosquitoes to hematophagy and consequently on their ability to transmit diseases. Altogether, these results may also contribute to the understanding of heme cell biology in eukaryotic cells.

## Introduction

Heme is a ubiquitous molecule involved in several cellular processes including signal transduction and transcriptional regulation. The intracellular concentration of heme is tightly controlled to prevent heme-driven cytotoxicity [1], which is frequently attributed to its capacity to promote oxidative stress [2]. Heme turnover is regulated through its degradation by heme oxygenases (HO), and the degradation products serve as antioxidants and signaling effectors [3]. Transcriptional regulation by heme is thus controlled by a feedback loop. Despite a well-documented involvement of heme in mammalian cell physiology and pathologies of immune-mediated inflammatory diseases [4,5], much less is known about the global transcriptional effects of heme on eukaryotic cells.

The digestion of hemoglobin inside the guts of blood-feeding organisms releases large quantities of heme, and several adaptations to ameliorate heme toxicity have been reported in these insect vectors [6–11]. Furthermore, ROS production in the midgut plays a key role in insect immunity through pathogen-killing [12,13]. In mosquitoes, ROS antagonize bacteria and *Plasmodium* infections [14,15]. Regardless these beneficial roles in pathogen clearance, ROS are potentially harmful to the host.

Present knowledge on heme modulation of gene expression is strongly biased towards its effect on cellular stress response and very few works are available on genome-wide effects of heme in gene transcription [16]. Here, we performed a transcriptome-wide analysis of heme influence on *A*. *aegypti* cells. Our results show that heme exposure leads to broad changes in expression of genes related to antioxidant activities, energy metabolism and cell cycle both *in vitro* and *in vivo*, revealing pleiotropic effects of heme on cell signaling. Furthermore, heme also modulates genes related to immunity, altering gut-resident microbiota and intricately influencing dengue virus replication in the mosquito. Thus, our data corroborate the role of heme molecule as gene expression regulator that influences *A*. *aegypti* physiology. The implications of these findings on the adaptation to hematophagy and pathogen transmission by *A*. *aegypti* are discussed.

## Results

### Heme has a unique and paramount role in transcript expression regulation

In order to study heme-induced global transcriptional changes, we performed a transcriptome-wide analysis of heme influence on the *A*. *aegypti* cell line Aag2. As heme-mediated effects on gene expression are thought to be related to oxidative stress, we compared the transcriptional profiles of Aag2 cells challenged with either 50 μM heme or 100 μM of the ROS inducer paraquat [17,18], using whole genome microarrays. The concentrations chosen to be used in the transcript expression assays imposed non-lethal stress to the cells (Figure A in S1 File).

**Fig 1:**
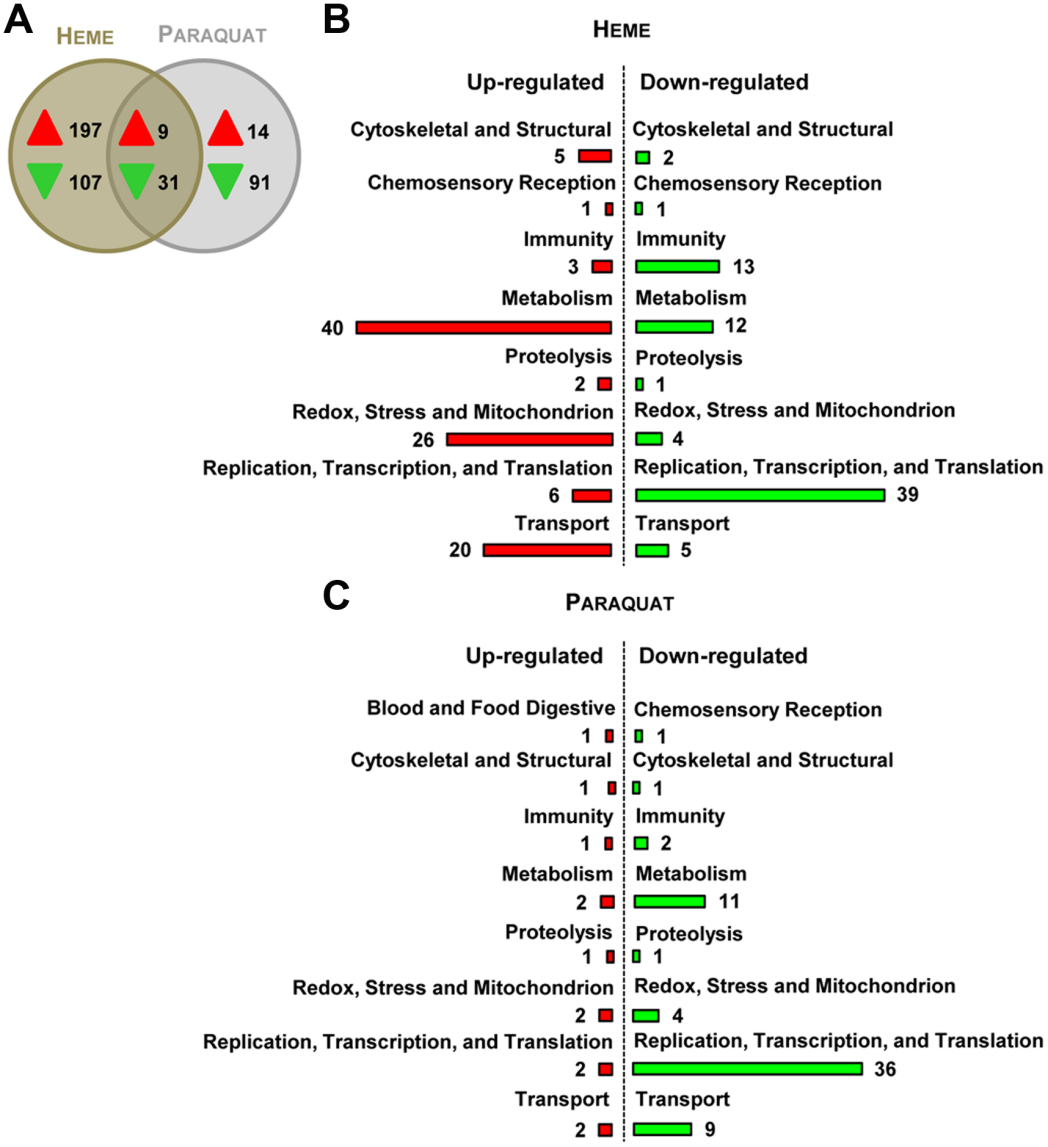
Heme and paraquat present different globall profiles in transcript expression regulation. (A) Venn diagram showing the numbers of unique and commonly regulated genes in heme- or paraquat- incubated cells compared with non-treated cells. The arrows indicate the direction of gene regulation. (B) Functional classification of significantly up-regulated or down-regulated genes caused by heme or (C) paraquat incubation. The bar plot represents the number of genes differentially expressed and functionally annotated. The cutoff value for the significance of gene regulation on these microarrays was 0.5 in log2 scale. The results were analyzed from four independent replicates. See also Figure B in S1 File and S1 Table.

Heme significantly regulated 344 transcripts in the cell line (206 induced and 138 repressed), whereas paraquat exposure resulted in the regulation of 145 transcripts (23 induced and 122 repressed) (Fig 1A). The heme-induced transcripts encoded typical antioxidant proteins such as ferritin, glutathione S-transferases (GSTs), cytochrome P450 and heat-shock proteins (S1 Table). A total of 9 and 31 transcripts were up- and down-regulated, respectively, by both challenges (Fig 1A), representing a common response to both stimuli that included transcripts associated with redox stress, metabolism, cell cycle control and transport-related proteins (Fig 1B and 1C and S1 Table). However, the most striking discovery was that ~70% (304 in 449 total) of transcripts were specifically regulated by heme, suggesting the existence of distinct signaling pathways. The differential expressions of 18 genes randomly chosen, was validated by quantitative real-time PCR, and they were consistent with those observed in the microarray-based assay (Figure B in S1 File).

Moreover, gene regulation by heme in Aag2 cell line was compared to data reported in the literature [19] on genome wide transcriptional profile from blood fed and sugar fed mosquito females (S2 Table). As expected, blood feeding altered expression of a larger set of genes compared to heme stimulus. However, most of the transcripts (~90%) regulated by heme were also differentially accumulated after a blood meal, suggesting that heme could be one of the blood components responsible for gene expression modulations observed *in vivo* (Figures C and D in S1 File).

### Blood meal-derived heme induces an antioxidant response in *A*. *aegypti* cells

**Fig 2:**
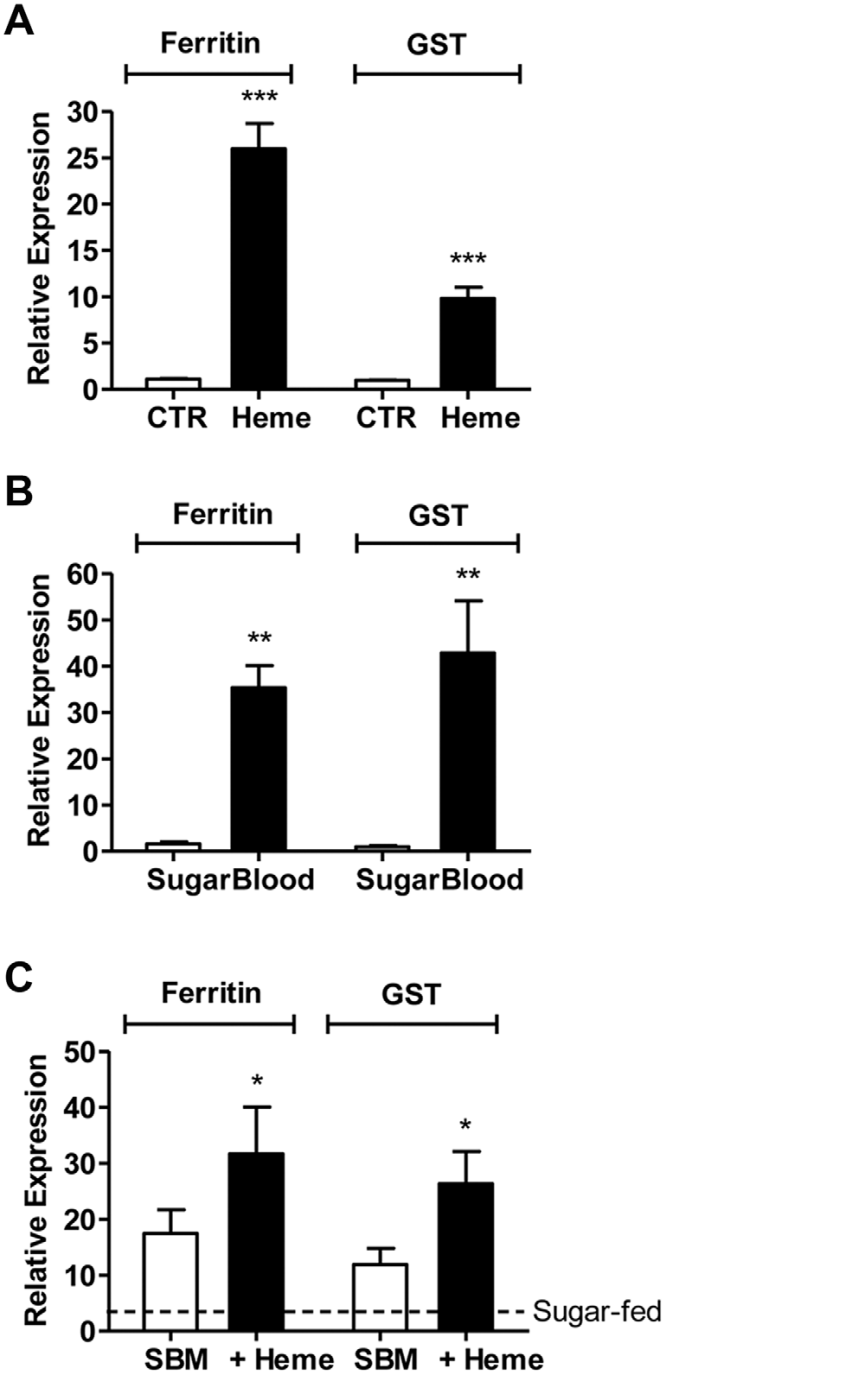
Heme- and blood-induced transcriptional changes are similar. Expression of the antioxidant genes ferritin and GST by real-time PCR in (A) Aag2 cells incubated for 24 hours with normal medium (control—CTR) or medium supplemented with 50 μM heme (Heme); (B) sugar-fed and blood-fed mosquito midguts at 24 h after feeding, and (C) mosquitoes fed with substitute blood meal (SBM) with or without 50 μM heme compared with sugar-fed mosquito midguts. Results are pools of at least 3 independent experiments. Error bars indicate the standard error of the mean. *** = p<0.001; ** = p<0.01; * = p<0.05 (by Student’s t-test).

*A*. *aegypti* undergo intense transcriptional changes after blood ingestion [19,20] and the midgut is extensively engaged to undertake protective responses, including the responses related to iron metabolism and stress [21]. Indeed, two of the most up-regulated genes, ferritin (heavy chain homologue (HCH) ferritin, ID: AAEL007385) and GST (GSTX2, ID: AAEL010500), were also stimulated by heme in the midguts of blood-fed females (Fig 2A and 2B), supporting the hypothesis that the modulation of redox gene expression after blood feeding is likely altered by heme release during digestion.

**Fig 3:**
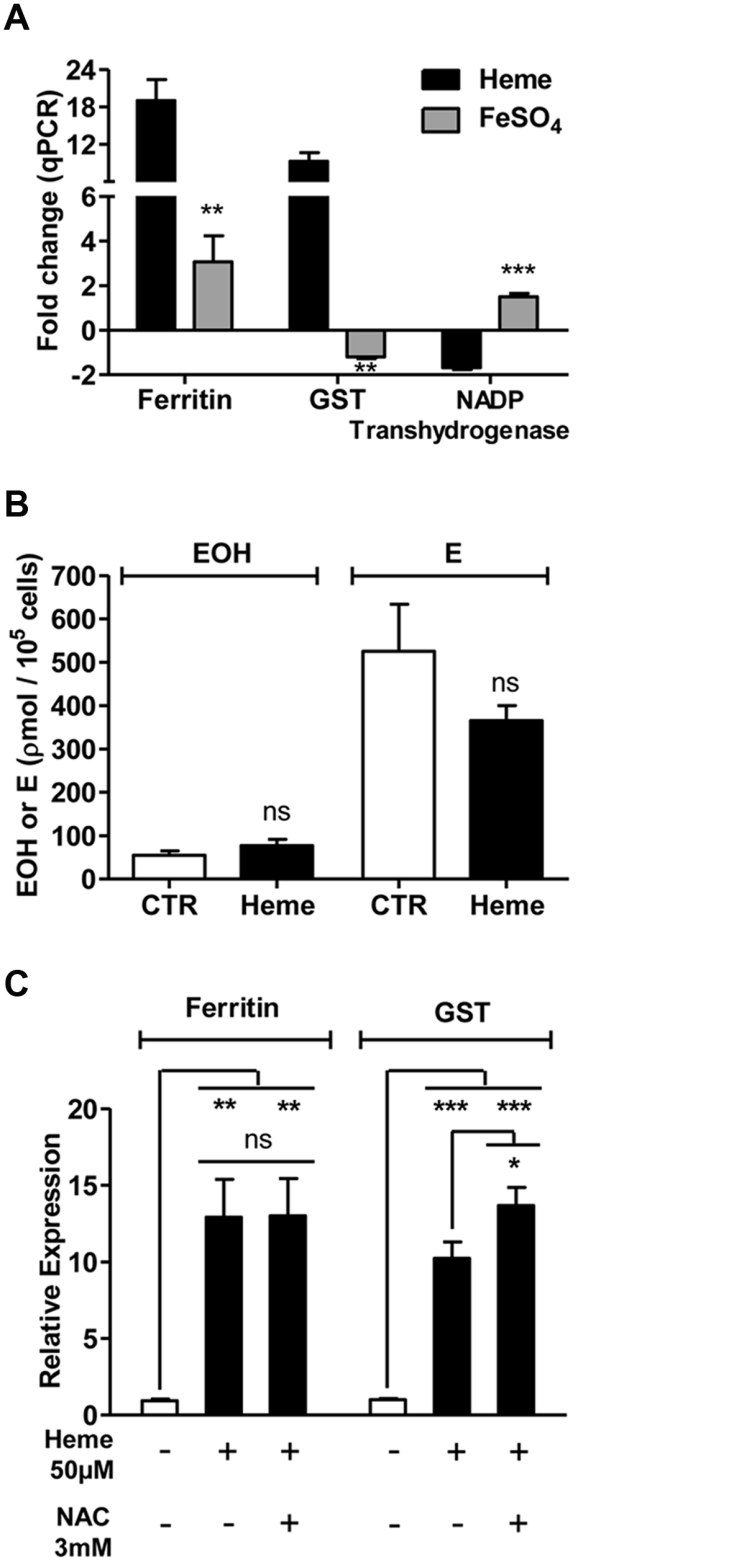
Heme-induced transcriptional changes are distinct from the effects induced by iron and are not related to changes in redox balance. (A) Comparison of the fold change in antioxidant gene expression induced by 50 μM heme and 50 μM ferrous sulfate in Aag2 cells as measured by qPCR. (B) Superoxide radical production in Aag2 cells after 6 h incubation with control medium and heme-supplemented medium measured by HPLC-separation of DHE oxidation products EOH– 2-hydroxyethidium; E—Ethidium. (C) The expression of antioxidant genes in Aag2 cells at 6 h of incubation with control medium or medium supplemented with 50 μM heme, with or without prior treatment with 0.5 mg/ml n-acetyl cysteine (NAC). The data represent at least 3 independent experiments. Error bars indicate the standard error of the mean. Ns = non-significant changes; *** = p<0.001; ** = p<0.01; * = p<0.05 (A and B—by Student’s t-test; C—by One-way ANOVA).

To separate heme-induced transcriptional changes *in vivo*, from the ones elicited by other components of host blood, we fed the insects with a chemically defined artificial diet (Substitute Blood Meal, SBM) supplemented with heme. The result clearly demonstrated that heme increased the mRNA levels of both ferritin and GST in the midgut of heme-fed females (Fig 2C). Moreover, heme-induced transcriptional regulation appears to act independently of changes in redox balance or iron (Fig 3). Therefore, heme appears to act by itself as a regulator of gene expression *in vivo*.

### Heme is an important regulator of energy metabolism and immune response

**Fig 4:**
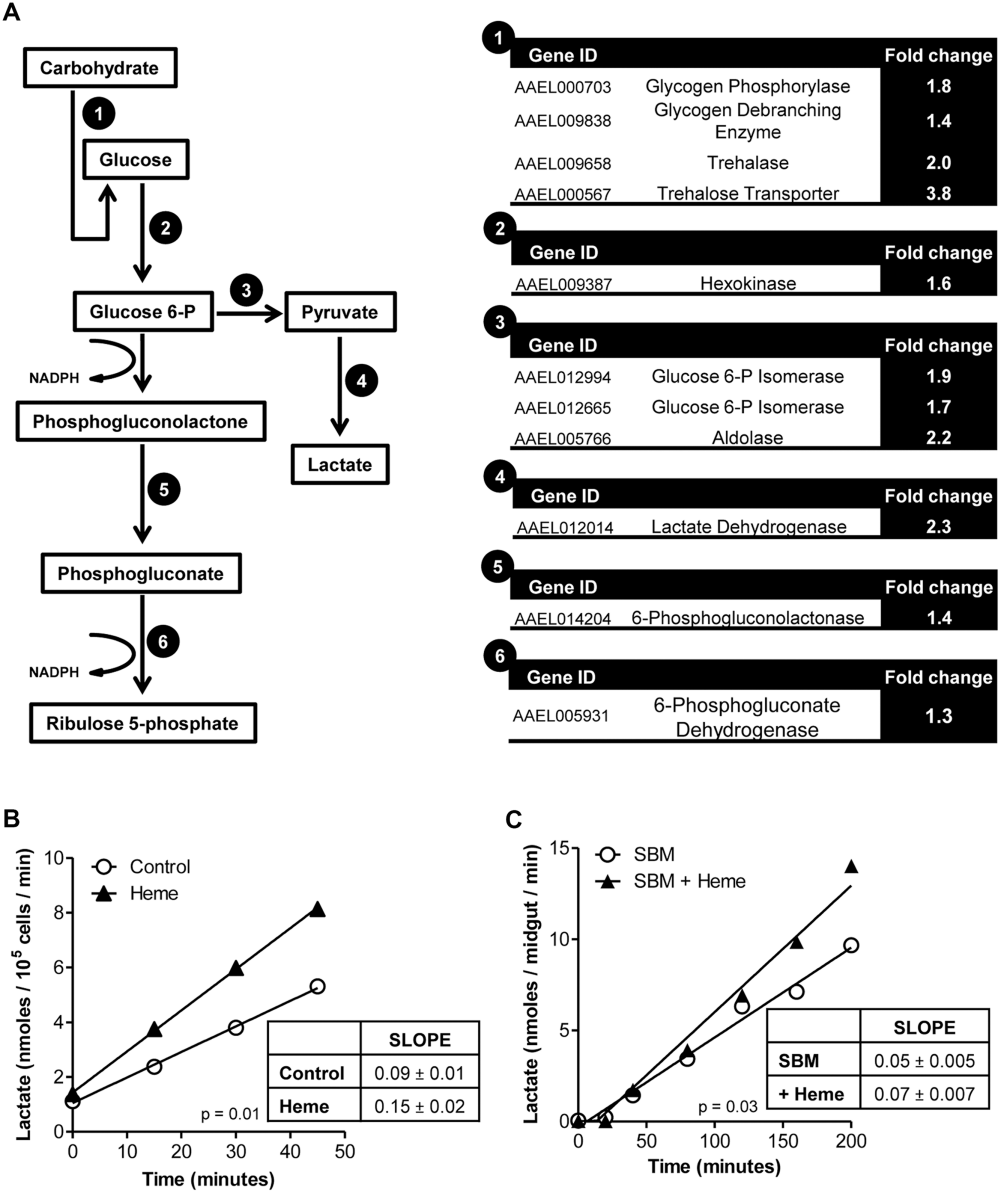
Aag2 cells undergo metabolic changes induced by heme. (A) Schematic figure representing the regulation of metabolic pathway genes by heme incubation from microarray data. Rate of lactate released in the medium of (B) 50 μM heme-incubated or control cells or (C) midguts of SBM (with or without heme)-fed females. At least 3 independent experiments are shown. The linear regression analysis performed showed a p-value<0.05, indicating that the differences between the slopes are significant.

Analysis of metabolism related-genes revealed that Aag2 cells exposed to heme had a higher transcript level of several genes involved in carbohydrate transport and mobilization, glycolysis, fermentation and the pentose phosphate pathway (PPP) (Fig 4A). To validate the observed transcriptional changes, we assessed the metabolic status of heme-exposed cells by monitoring changes in lactate production. The results show that the incubation significantly increased the rate of lactate production by 1.7-fold when compared with control cells (Fig 4B). Midgut lactate release was also increased (1.4-fold) in heme-fed mosquitoes, reinforcing the physiological relevance of these findings *in vivo*.

**Fig 5:**
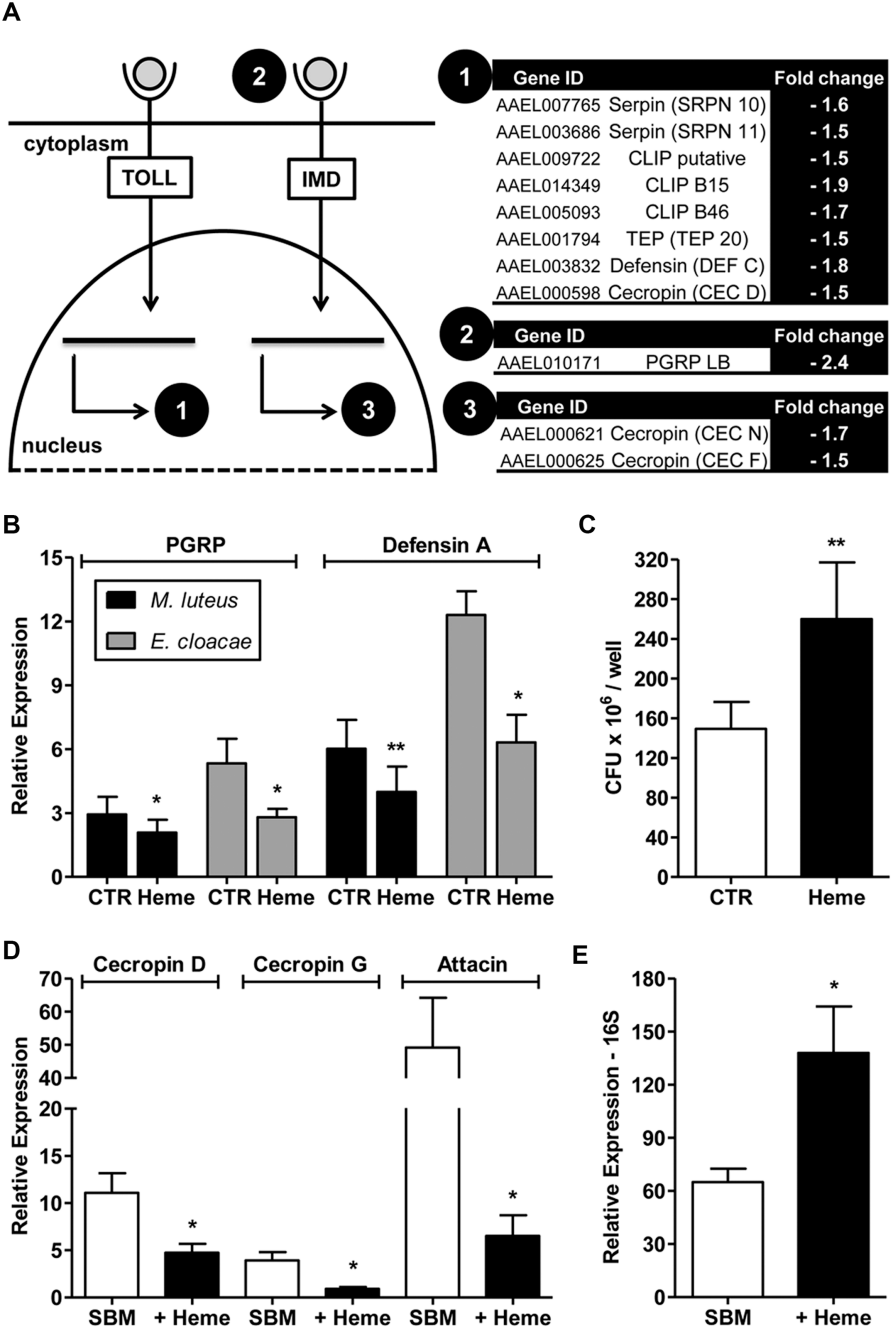
The immune response is compromised by heme supplementation *in vitro* and *in vivo*. (A) Schematic figure representing the regulation of immune pathway genes by heme incubation from microarray data. (B) Aag2 cells were incubated with vehicle (control—CTR) or 50 μM heme and then challenged with heat-killed *M*. *luteus* or *E*. *cloacae*. The bar charts show the defensin and peptidoglycan recognition protein gene expression levels relative to the ribosomal protein Rp49 and normalized by a non-challenged sample as measured by qPCR. (C) Gram-negative (*E*. *cloacae*) bacteria were inoculated into the cell culture medium in 24-well plates containing Aag2 cells pre-incubated with vehicle or 50 μM heme. The graph shows CFU numbers as a measure of bacterial growth after 3 h incubation at 28°C. (D) Evaluation of immune gene expression in orally infected (*S*. *marcescens*) mosquitoes fed with substitute blood meal (SBM) with or without 50 μM heme. (E) Culture-independent evaluation of midgut natural microbiota in unchallenged mosquitoes fed with SBM with or without 50 μM heme through qPCR for bacterial ribosomal 16S RNA normalized by a control sample (sugar-fed). At least 4 independent experiments are shown. Error bars indicate the standard error of the mean. ** = p<0.01; * = p<0.05 (by Student’s t-test).

Further analysis of the transcriptome data showed that the abundance of several immune-related transcripts was significantly decreased in response to heme exposure (Fig 5A). Hence, we hypothesized that heme-exposed cells might be more susceptible to bacterial challenge. Challenging Aag2 cells with heat-killed Gram-positive (*Micrococcus luteus*) or negative (*Enterobacter cloacae)* bacteria resulted in a substantial induction of peptidoglycan recognition protein (PGRP) and defensin genes as measured by qPCR. However, in heme pre-exposed cells, this induction promoted by bacterial challenge was significantly lower (Fig 5B).

Aag2 cells are immune-competent and capable of limiting the growth of bacteria through phagocytosis and the secretion of antimicrobial peptides (AMPs) [22,23]. We tested whether the reduced production of immune response effectors in heme-incubated cells would result in lower anti-bacterial activity. The cells were pre-incubated with heme and then challenged with live bacteria (*E*. *cloacae*). *E*. *cloacae* co-cultured with heme-incubated cells had higher growth when compared with the control samples (Fig 5C), confirming that the down-regulation of immune genes by heme resulted in an impaired immune response.

### Heme influences the *in vivo* immune response and gut microbiota control

To test whether heme acts as an immunomodulator *in vivo*, we evaluated immune-related gene expression and the changes in gut microbiota of heme-fed mosquitoes. Immune genes such as cecropin D, cecropin G and attacin showed strongly diminished expression in the midguts of heme-fed mosquitoes orally challenged by *Serratia marcescens* (a gram-negative bacteria) (Fig 5D). We subsequently investigated the effect of this immune inactivation on mosquito gut bacterial load. Heme supplementation resulted in ~2.0-fold increase in the microbial 16S rRNA levels (Fig 5E). This result clearly shows that heme directly influences the immune response also *in vivo*, which results in the proliferation of the midgut microbiota.

### Heme affects mosquito susceptibility to DENV infection

**Fig 6:**
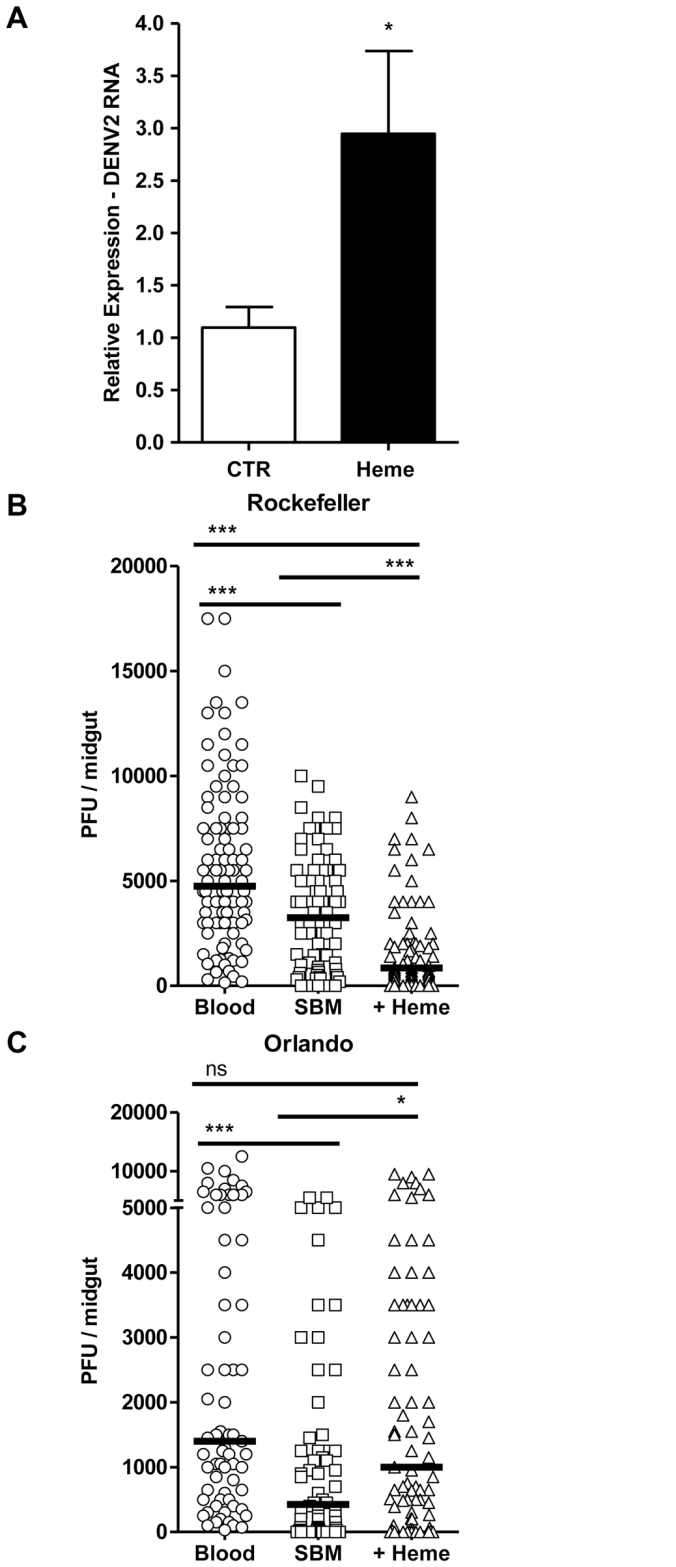
Heme alters dengue virus replication *in vitro* and *in vivo*. (A) Aag2 cells were incubated with vehicle (CTR) or 50 μM heme 6 h prior to DENV2 infection. The graph shows viral RNA levels after 4 days of infection as determined by qPCR. At least 4 independent experiments are shown. Error bars indicate the standard error of the mean. * = p<0.05 (by Student’s t-test). Midgut DENV2-NGC titers at 7 days post blood meal in (B) Rockefeller (susceptible) and (C) Orlando (refractory) mosquitoes. Data are a pool of at least 3 independent biological replicates. The statistical significance values were determined using the Kruskal-Wallis test with Dunn’s post-test (* p<0.05, ** p<0.01, and *** p<0.001).

To assess the impact of heme exposure on Aag2 cell susceptibility to dengue virus (DENV) infection, we quantified DENV2 RNA in the supernatant of Aag2 cells pre-incubated or not with heme. DENV2 RNA levels in heme-incubated cells were significantly higher compared with the controls (Fig 6A).

We found a differential impact of heme on DENV2 infection of the midgut of DENV susceptible or refractory mosquito strains. Feeding the susceptible *A*. *aegypti* strain (Rockefeller) on a DENV2-supplemented SBM diet resulted in significantly lower midgut DENV2 titers compared to delivering the virus through a regular blood meal at 7 days post infection. Providing the virus through a heme-supplemented SBM resulted in even lower midgut DENV2 titers (Fig 6B). By contrast, feeding DENV refractory mosquitoes (Orlando) on a heme-supplemented SBM resulted in increased midgut DENV2 titers, similar to the levels observed for mosquitoes fed on the virus through a normal blood meal. This finding indicates that heme facilitates DENV infection in the refractory but not in the susceptible mosquito strain fed on a heme-supplemented SBM (Fig 6C).

## Discussion

Despite its essential function in all aerobic organisms, the effects of heme on gene expression are yet poorly studied and are strongly biased towards the role of heme in stress-responses. Furthermore, oxidative stress has largely been considered a single dimensional imbalance of oxidants and antioxidants. However, over the last decade, the conceptual framework of free radical biology has evolved from a toxicological view towards a broader definition of oxidative stress as a perturbation of cell signaling mediated by oxidants [24]. Most of the literature on heme cell biology still does not fully acknowledge this change; therefore, heme is still referred to as a promoter of oxidative damage and its effects on cell homeostasis are frequently reported as reactions to an oxidant insult. In the blood-feeding *A*. *aegypti* dengue vector, heme release during digestion is coupled with several anti-cytotoxic adjustments [25] and has recently been linked to sensing the availability of a blood meal for egg development [26]. To gain more comprehensive insight into heme’s effect on mosquito physiology at the molecular level, we characterized the global transcriptional regulation promoted by heme in *A*. *aegypti*. We found that heme acts as a pleiotropic and effective signaling molecule, influencing mosquito adaptation to blood digestion and pathogen infection.

We compared the transcriptomes of cells exposed to heme or the pro-oxidant paraquat. As expected, both stimuli up-regulated several antioxidant genes. An exception is the heme-degrading enzyme heme oxygenase (HO) that is transcriptionally induced by heme in nearly all cell types studied. However, our result is consistent with the *in vivo* observation from previous genome-wide studies that also did not find increase in HO transcript level after a blood meal in *A*. *aegypti* [19,20]. This is an interesting finding that may represent an adaptation to hematophagy and deserves further investigations.

Transcriptome responses to heme- or paraquat-exposure had limited overlap; most of the regulated genes were exclusively modulated by only one of these compounds, suggesting an existence of heme-specific signaling pathways in the mosquito. Among the commonly-regulated transcripts, those with functional roles in thiol stress and chaperones processes displayed the greatest extent of regulation. Several heme-induced transcriptional changes may account for specific adaptations that attenuate heme toxicity, such as the up-regulation of ABC (ATP-binding cassette) transporter expression (S1 Table). Indeed, recently ABC transporters have been implicated in mediating heme export in *Caenorhabditis elegans* [27]. Also, one of top up-regulated transcripts in heme-incubated cells is the one encoding for GSTX2 (AAEL010500) (S1 Table), which has been shown to bind heme and has a suggested role in protecting mosquitoes against heme toxicity after a blood meal [28].

The transcriptional changes induced by paraquat were similar to reports obtained with other cell types and organisms. Paraquat-exposure in *Drosophila melanogaster* through feeding elicits a stress-related response, similar to the responses observed during aging and general cellular stress [29,30]. Our data revealed a comparable pattern; several cell cycle control and general stress-related genes were altered by paraquat exposure (Fig 1C). Conversely, the overall heme-induced changes were rather unique and more complex than those found in the paraquat-exposed cells. Previous studies on the cell biology of heme have suggested that heme-induced gene expression is mediated by oxidative stress. By contrast, our assays showed that ROS levels were not increased upon sub-lethal (Figure A in S1 File) concentrations of heme exposure, although several stress genes were up-regulated, suggesting that a change in redox balance is unlikely to play a major role in the control of the heme-associated response described in our study (Fig 3). Moreover, it suggests that the mosquito response to heme is directed to prevent oxidative stress, rather than being a reaction to it. In fact, it has been shown that after blood feeding, *A*. *aegypti* shuts down ROS generation in the gut as a strategy to avoid heme-mediated oxidative stress [31]–this phenomenon may reflect a first line of defense against the potential harmful effects of heme that culminate with the enhanced expression of mosquito stress genes (Fig 2).

Heme exposure of *A*. *aegypti* cells appears to induce a metabolic adaptation that may enhance glucose uptake and usage through transcriptional regulation (Fig 4A), which is phenotypically evidenced by an increase in lactate production of either heme-incubated cells or midguts of heme-fed mosquitoes (Fig 4B and 4C). Furthermore, heme up-regulates genes encoding enzymes involved in the oxidative branch of PPP (Fig 4A), which may account for the need of NADPH as a provider of reducing equivalents for the thiol-based antioxidant defenses. In fact, in yeast, a shift in primary carbon metabolism is the fastest response to oxidative stress, and an important link exists between the activity of the PPP and the transcriptional antioxidant response [32]. Additionally, mammalian cells with the Keap1/Nrf2 pathway activated, a molecular sensor of redox challenge, have increased glucose uptake and higher activity of PPP [33]. We propose that, in *A*. *aegypti*, heme may be a trigger for metabolic adaptation, which may help prevent an oxidative burst. Related metabolic changes have also been observed in mosquitoes [34] and in several other blood-feeding organisms [35] in response to a blood meal.

Heme-mediated effects on gene expression appear to go alongside with previous studies on the global transcriptional regulation that mosquitoes undergo after a blood meal. Bonizzoni *et al*. (2011) showed that the pattern of expression detected in blood-fed *A*. *aegypti* females, at the whole-body level, includes several genes whose products are involved in detoxification pathways, energy metabolism, immunity, as well as those implicated in specific cellular responses such as transport and cell cycle regulation. In a comparative analysis, we could find that ~90% of the total genes regulated through heme stimulus in Aag2 cells are also differentially accumulated after a blood meal in the mosquito. Inside the shared response, the majority of the enriched transcripts in heme-incubated cells are commonly up-regulated in blood-fed females. Also, many genes whose levels decrease after a blood meal are also found to be down-regulated upon heme incubation in Aag2 cells (Figure C in S1 File and S2 Table). This common response is especially true for the pattern of expression of individual genes involved in the main cellular processes discussed here (Figure D in S1 File). Among the transcripts that show a common up regulation are glutathione S-transferases, heat-shock proteins and many genes involved in glycolysis and PPP. Moreover, in blood-fed females, as in heme-incubated cells, genes encoding immune receptors and effectors showed decreased transcript accumulation (S2 Table). These correlative effects suggest that heme could account for a significant part of the complexity of the blood-induced transcriptional changes in mosquitoes.

Likewise, Sanders et al. (2003) performed microarray analyses using samples from midguts of blood-fed females and found a group of differentially expressed genes that had impressively common functions with the transcripts regulated upon heme-incubation of Aag2 cells, described for thehere. This local tissue response include an increase in gene transcripts involved in detoxification, stress signaling, and metabolism, as well as decreases in gene transcripts involved in translation initiation. In an individual comparative analysis, we found specially that genes encoding ABC transporters, ferritin, catalase, hexokinase, heat shock 70, all of which are proposed to be part of a heme-induced response, were up-regulated 24 hours after blood meal in the midgut. Collectively, these transcription patterns illustrate that, during blood digestion, midgut cells could undergo similar transcriptional changes as the Aag2 cell line upon heme incubation. Despite the fact that an embryonic cell line does not represent an unique adult tissue, this correlation clearly shows that regardless of the intricate physiological adaptations that follow a blood meal, the use of the cell line as a biological tool for the assessment of specific cellular processes is valid and could, in part, represent what is observed *in vivo*.

Nevertheless, several heme-related changes are more complex and appear to have a further direct role in vectorial competence of the mosquito. Overall, genes comprising the main immune pathways in the mosquito are down-regulated by heme, and this effect weakens the insect immune response against bacteria (Fig 5). In fact, it has been previously demonstrated that blood-fed mosquitoes have reduced expression of immune genes [19]. Data presented here suggest that heme may have an important role in this feeding-induced immunosuppression. Oliveira et al. [31] revealed a heme-permissive effect on microbial growth in the *A*. *aegypti* gut. This increase in gut-resident bacteria, which was also demonstrated in our study (Fig 5E), may be due to decreasing pathogen-killing ROS levels as previously stated; however, this increase might be additionally due to a direct role of heme in the immune pathways that encode for pathogen recognition proteins and AMPs.

The results obtained here also suggest that, in addition to these effects on immune response and microbiota, heme influences viral replication. In Aag2 cells viral replication was enhanced by heme incubation (Fig 6A); however, our data show that the role of heme on mosquito immune responses *in vivo* is more intricate and depends on the genetic background of mosquitoes. The susceptible mosquito strain (Rockefeller) showed lower midgut DENV titers upon heme feeding (Fig 6B). This result contradicts the findings observed using the *in vitro* Aag2 cell system. *A*. *aegypti* susceptibility to DENV2 infection could be affected by heme in two different ways. First, heme increases the microbial load in the mosquito midgut, which has an antagonistic effect on viral infection [36,37]. In this case, the lowering of viral infection observed in the susceptible strain reflects a heme indirect effect, triggered by its primary result on basal levels of midgut microbiota (Fig 5E). Second, heme directly suppresses the mosquito immune activity; however, a role of heme as an immunosupressor appears to be dependent on a robustly active immune system. Previous studies [38] showed that the Rockefeller mosquitoes already have low immune activity; therefore, heme supplementation may have stronger effects on the microbial load, finally resulting in lower DENV2 virus titers in the Rockefeller mosquito midguts. In contrast to the susceptible strain, heme-fed refractory mosquitoes (Orlando) showed a higher susceptibility to viral infection (Fig 6C). In comparison with the Rockefeller-susceptible strain, basal abundance levels of several immunity-related transcripts are higher in the Orlando mosquitoes [38]. Hence, the heme-induced down-regulation of immune pathways such as Toll and IMD (S1 Table) could have a stronger effect on a potent immune system, which may result in the enhanced susceptibility observed in the Orlando strain. Another plausible explanation comes from the comparison of transcriptional changes that take place in cells exposed to heme, present in this report, with transcriptome data from blood fed mosquitoes (Figures C and D in S1 File and S2 Table), that clearly showed that the heme signal is a part of a signaling network that is operating after a blood meal. Certainly, other important regulatory pathways are acting together with heme, and thus, the data presented here allow us to hypothesize that the interaction between heme-induced pathways and these other blood meal induced regulatory circuits could account for the strain-specific effect of heme on DENV proliferation in intact mosquitoes.

Notably, in mammals it has been shown that heme is a complex modulator of immune response, with both pro-inflammatory and anti-inflammatory effects depending on the cellular context and exposure time. Short-term pro-inflammatory effects have been associated to exposure of neutrophils and macrophages to heme [39]. Several immune-mediated inflammatory diseases are associated with the accumulation of free heme, which induces programmed cell death in response to pro-inflammatory agonists, increasing tissue damage [40,41]. On the other hand, heme-induced regulation of HO is associated with cytoprotective effects, mostly due to the heme catabolism end products such as biliverdin and carbon monoxide. Therefore, we propose that the observed strain-specific heme effect may be due to a differential response of these *A*. *aegypti* strains, associated to their genetic background and to the temporal window of observation.

It is well established in vector literature that the midgut is the site of complex interactions that include host vector immune defenses, vertebrate blood factors, the pathogen (virus or parasite), and the symbiotic microbiota. Indeed, Pakpour et al. [42] recently highlighted a number of blood-derived factors that added further complexity to vector-borne pathogen transmission. In this study, we showed that heme is a blood-related factor that is involved in the regulation of vector competence; however, it remains unclear how heme directly affects mosquito immune pathways that acts against viruses and bacteria, and this question will be addressed in future research. Prospective studies will also target a better understanding of molecular mechanisms that underlie specific gene regulation induced by heme.

Here we provide a novel view of heme signaling, challenging its conventional role as a sole oxidative stressor molecule, with an influential impact on eukaryotic cell biology of heme. Our study also implicates heme signaling as a key element in mosquito adaptation to blood ingestion and with major implications to vectorial competence. This important heme feature deserves to be investigated in other blood-feeding vectors.

## Materials and Methods

### Ethics statement

All animal care and experimental protocols were approved by the institutional care and use committee (Comitê para Experimentação e Uso de Animais da Universidade Federal do Rio de Janeiro/CEUA-UFRJ) and the NIH Guide for the Care and Use of Laboratory Animals (ISBN 0–309-05377-3). The protocols were approved under the registry CEUA-UFRJ #155/13.

### Mosquitoes rearing and cell culture maintenance

*A*. *aegypti* (Red Eye strain) were raised in a mosquito-rearing facility at the Federal University of Rio de Janeiro, under a 12 h light/dark cycle at 28°C and 70–80% relative humidity. The adults were maintained in a cage and given a 10% sucrose solution *ad libitum*. Four to seven day-old females were used in the experiments. For the DENV experiments, the mosquitoes were reared at the Johns Hopkins Malaria Research Institute. The DENV-susceptible *A*. *aegypti* strain (Rockefeller/UGAL) and DENV-refractory strain (Orlando) [38] were used to test the effect of heme on DENV infection. The mosquitoes were maintained in a similarly controlled environment.

*A*. *aegypti* Aag2 cells [43] were maintained at 28°C in Schneider´s *Drosophila* medium with L-glutamine (Invitrogen-GIBCO, Carlsbad, CA, USA) supplemented with 10% fetal bovine serum (FBS) (Cultilab, Campinas, SP, Brazil). At 80–90% confluence, the cells reached a density of ~2x10^5^ cells/cm^2^.

### Mosquito meals and infection

The mosquitoes were artificially fed with the following different diets: (1) 10% sucrose (*ad libitum*), (2) heparinized-rabbit blood or (3) Substitute Blood Meal (SBM) with or without 50 μM heme. For the evaluation of immune gene expression, the female mosquitoes were orally infected with living *S*. *marcescens* (gram-negative bacteria) (~10^9^ bacteria/ml). The assessment of midgut bacterial growth was performed through qPCR for bacterial ribosomal 16S RNA, given that not all bacteria from natural midgut flora are cultivable. For RNA sample preparation, the female midguts were dissected 24 h after feeding.

To test the effect of heme on DENV infection, SBM was used for DENV feeding to mimic the natural route of viral infection. The fed mosquitoes were maintained at the normal rearing conditions for 7 days before midgut dissection to determine midgut DENV titers. The virus (New Guinea C strain, DENV2) was propagated in the C6/36 cell line following a protocol previously described [37].

The SBM solution is based on a previously formulated protein meal [44,45] composed of 116 mg/ml BSA; 30 mg/ml λ-globulin and 2 mg/ml cholesterol in Tyrode Buffer with or without 50 μM heme.

### DENV titration

The virus (New Guinea C strain, DENV2) was added to the feeding solution. Normal blood meal was used as a positive control group. To determine midgut DENV titers, individual mosquito midguts were homogenized, 10-fold serially diluted, and then inoculated onto 80% confluent BHK cells in 24-well plates. The plates were rocked for 15 min at room temperature and then incubated for 45 min at 37°C and 5% CO_2_. Subsequently, 1 ml of DMEM containing 2% heat-inactivated FBS, 1% L-glutamine, 10 unit/ml Penicillin, 10 μg/ml Streptomycin, 5 μg/ml Plasmocin, and 0.8% methylcellulose was added to each well, and the plates were incubated for 6 days at 37°C and 5% CO_2_. The plates were fixed and stained with a 1% Crystal violet in methanol/acetone mixture (1:1 volume) for at least 1 h at 4°C.

### Cell treatment and DENV infection

Before each experiment, the cells were incubated with the heme solution diluent (0.1 M NaOH, 0.01 M phosphate buffer, used as control), 50 μM Heme (Hemin, Frontier Scientific Inc., Logan, UT, USA), or 100 μM paraquat (ChemService, West Chester, PA, USA), and all solutions used were sterile.

For bacterial challenges, after pre-incubation with 50 μM heme, the cells were incubated with two different heat killed bacteria as previously described [46]: *M*. *luteus*, a Gram positive bacteria, or *E*. *cloacae*, a Gram negative bacteria (~10^2^ bacteria/cell). The cell lysates were harvested at 4 h after bacterial challenge for RNA extraction. In the bacterial growth experiments, after pre-incubation with 50 μM heme, the cells were co-cultured with living *E*. *cloacae* at 1 bacterium per cell proportion for 3 hours. Each cell sample supernatant was diluted and submitted to bacterial growth on 1.5% LB Agar plates at 37°C overnight.

For viral infection, the cells were infected with DENV2 (Halsted 16686) using a MOI (multiplicity of infection) of 1. The cells were incubated in the presence of virus in Schneider´s *Drosophila* medium without FBS for 1 hour at 28°C. Next, medium supplemented with 10% FBS was replaced in all wells. Cells were incubated for 4 days at 28°C.

### Cell viability assay

For those assays the cells were grown on 24 well plates. 24 h after plating, cells were incubated with different concentrations of Heme (10–500 μM) or Paraquat (10–2000 μM) for additional 24 h. After each treatment, cell viability was accessed using MTT assay, as described previously [47]. Results were analyzed using a statistical software package (GraphPad Prism 5).

### RNA extraction and qPCR analysis

For the qPCR assays, the RNA of the midgut or cell samples was extracted using TRIzol (Invitrogen, Carlsbad, CA, USA) according to the manufacturer’s protocol. Complementary DNA was synthesized using the High-Capacity cDNA Reverse transcription kit (Applied Biosystems, Foster City, California, USA). The qPCR was performed with the StepOnePlus Real Time PCR System (Applied Biosystems, Foster City, CA, USA) using the Power SYBR-green PCR master MIX (Applied Biosystems). The Comparative Ct Method [48] was used to compare the changes in the gene expression levels. The *A*. *aegypti* ribosomal protein 49 gene (Rp49) was used as an endogenous control, based on previous data [49]. Results were analyzed using a statistical software package (GraphPad Prism 5). All oligonucleotides sequences used in qPCR assays are available in the S1 File.

### Microarray gene expression analysis

Following the previous stated experimental and control conditions, cells were submitted to total RNA extraction using the Qiagen RNeasy Mini Kit (Qiagen Inc., Valencia, CA, USA) according to the manufacturer’s protocol. Two micrograms of total RNA were used for probe synthesis of cy3- and cy5-labeled cRNA. The hybridizations were conducted on an Agilent-based microarray platform using custom-designed whole-genome 8 x 60K *A*. *aegypti* microarrays, and the arrays were scanned with an Agilent Scanner (Agilent Technologies Inc., Santa Clara, CA, USA). The probes representing the same gene had their signals averaged to a single expression value per gene. The gene expression values were then analyzed using the Significance Analysis of Microarrays (SAM) method (*samr* R package) [50]. The enriched KEGG pathways were analyzed with KOBAS [51] and guided manual analyses were described here. The numeric microarray gene expression data is presented in S1 Table.

### Kinetics of lactate release in the culture media

The lactate release assay was performed as previously described [52]. Briefly, after 18 h of control or heme incubation, the Aag2 cell culture medium was replaced by fresh RPMI 1640; after 24h of SBM (with or without heme) feeding, mosquitoes midguts were dissected and incubated in a 48-well plate, also in RPMI 1640. The supernatant aliquots were collected every 15 (Aag2) or 20 (midguts) minutes and incubated in a hydrazine/glycine buffer (pH 9.2), containing 5 mg/ml β-NAD^+^ and 15 units/ml lactate dehydrogenase (LDH). The absorbance at 340 nm was monitored in a microplate reader (SpectraMax M5, Molecular Devices, Sunnyvale, CA, USA) and was correlated with the presence of lactate on samples from a standard curve [53]. Results were analyzed using a statistical software package (GraphPad Prism 5).

## Supporting Information

S1 File **(Figure A)** Aag2 cell viability upon heme or paraquat incubation—cells were grown in the absence (CTR) or presence of various concentrations of Heme (A)–the zero “0” concentration correspond to the heme diluent solution—or Paraquat (B) for 24 h. After this incubation time, cell viability was analyzed by MTT assay. Arrows indicate the concentrations used for further gene expression assays—which do not impose cell mortality greater than 20%. Values represent mean ± SEM; N = 3. **(Figure B)** Validation of microarray results—microarray data were verified by qRT-PCR of RNA from 18 genes of cells incubated with heme or paraquat. RNA from control cells was used as the reference sample. Data were compared with internal Rp49 control and the fold change was obtained using the 2^(-ΔΔCt)^ method. *As determined by the significance (p<0*.*0001) and the Pearson’s correlation coefficient*, *the qPCR results were consistent with the changes observed in the microarray*. *The significance was determined using GraphPad Prism (v*. *5)*. **(Figure C)** Analysis of the number of transcripts regulated in both heme-incubated Aag2 cells and blood-fed *Aedes aegypti* females—Venn diagram illustrating the numbers of unique and commonly regulated genes in heme-incubated cells and blood-fed females. Among those common transcripts, several were up- or down-regulated in both assays or in only one of them. *Data originally found in: Bonizzoni *et al*. (2011) BMC Genomics 12:82. **(Figure D)** Functional quantitative analysis of transcripts regulated in both heme-incubated Aag2 cells and blood-fed *Aedes aegypti* females—Venn diagrams illustrating the numbers of unique and commonly regulated transcripts in heme-incubated cells and blood-fed females. Top to bottom panels represent data of transcripts functionally associated to immunity, metabolism, or redox, stress and mitochondrion processes, respectively. *Data originally found in: Bonizzoni *et al*. (2011) BMC Genomics 12:82.
(DOCX)

S1 Table Fold change values and functional groups of transcripts that were enriched or depleted in heme- or paraquat-incubated cells. Functional group abbreviations: CS, cytoskeletal and structural; CSR, chemosensory reception; DIV, diverse functions; DIG, blood and sugar food digestive; IMM, immunity; MET, metabolism; PROT, proteolysis; RSM, redox, stress and mitochondrion; RTT, replication, transcription, and translation; TRP, transport; UKN, unknown functions.
(XLSX)

S2 Table List of all the transcripts regulated in both heme-incubated Aag2 cells and blood-fed *Aedes aegypti* females. The blood-fed transcription data was extracted from then RNA-seq library found in Bonizzoni *et al*. (2011). Transcripts were analyzed according to its pattern of expression—up (+) or down (-) regulated—in both *in vitro* and *in vivo* assays, in comparison to control cells or sugar-fed females, respectively. The main transcripts discussed in the text are highlighted in yellow. Functional group abbreviations are stated above.
(XLSX)
