# Supplementary material for: Heme Signaling Impacts Global Gene Expression, Immunity and Dengue Virus Infectivity in Aedes aegypti
Source: PLoS One. 2015 Aug 14;10(8):e0135985. doi: 10.1371/journal.pone.0135985 (PMC4537099; doi:10.1371/journal.pone.0135985)
Supplement: S1 File — (Figure A) Aag2 cell viability upon heme or paraquat incubation—cells were grown in the absence (CTR) or presence of various concentrations of Heme (A)–the zero “0” concentration correspond to the heme diluent solution—or Paraquat (B) for 24 h. After this incubation time, cell viability was analyzed by MTT assay. Arrows indicate the concentrations used for further gene expression assays—which do not impose cell mortality greater than 20%. Values represent mean ± SEM; N = 3. (Figure B) Validation of microarray results—microarray data were verified by qRT-PCR of RNA from 18 genes of cells incubated with heme or paraquat. RNA from control cells was used as the reference sample. Data were compared with internal Rp49 control and the fold change was obtained using the 2(-ΔΔCt) method. As determined by the significance (p<0.0001) and the Pearson’s correlation coefficient, the qPCR results were consistent with the changes observed in the microarray. The significance was determined using GraphPad Prism (v. 5). (Figure C) Analysis of the number of transcripts regulated in both heme-incubated Aag2 cells and blood-fed Aedes aegypti females—Venn diagram illustrating the numbers of unique and commonly regulated genes in heme-incubated cells and blood-fed females. Among those common transcripts, several were up- or down-regulated in both assays or in only one of them. *Data originally found in: Bonizzoni et al. (2011) BMC Genomics 12:82. (Figure D) Functional quantitative analysis of transcripts regulated in both heme-incubated Aag2 cells and blood-fed Aedes aegypti females—Venn diagrams illustrating the numbers of unique and commonly regulated transcripts in heme-incubated cells and blood-fed females. Top to bottom panels represent data of transcripts functionally associated to immunity, metabolism, or redox, stress and mitochondrion processes, respectively. *Data originally found in: Bonizzoni et al. (2011) BMC Genomics 12:82. (DOCX) [file pone.0135985.s001.docx]

**
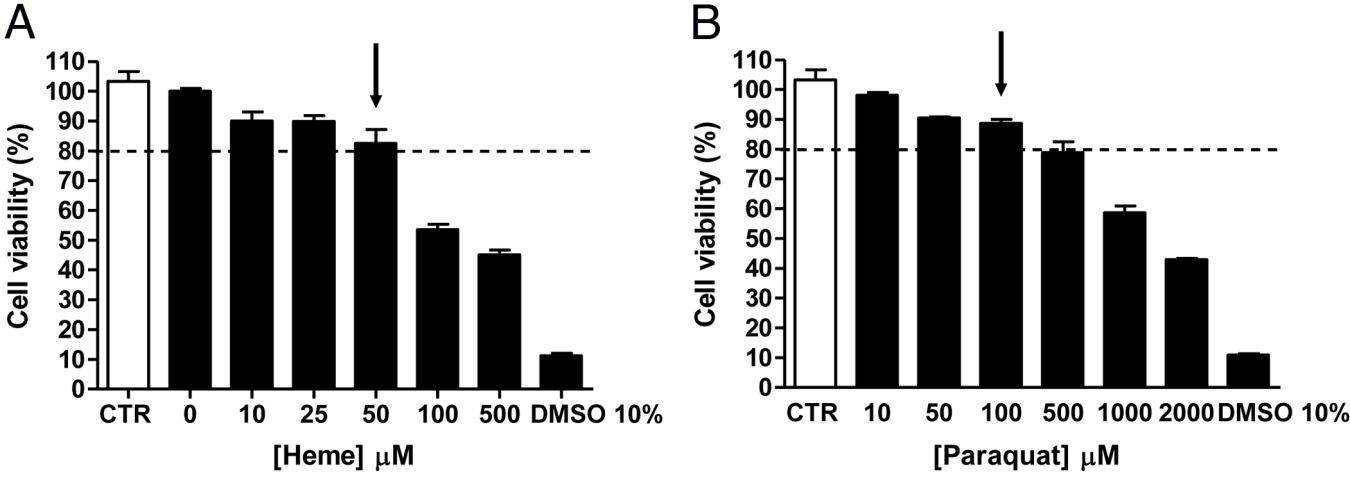
**

**FIGURE A: Aag2 cell viability upon heme or paraquat incubation.**

Cells were grown in the absence (CTR) or presence of various concentrations of Heme (A) – the zero “0” concentration correspond to the heme diluent solution – or Paraquat (B) for 24 h. After this incubation time, cell viability was analyzed by MTT assay. Arrows indicate the concentrations used for further gene expression assays – which do not impose cell mortality greater than 20%. Values represent mean ± SEM; N=3.

**
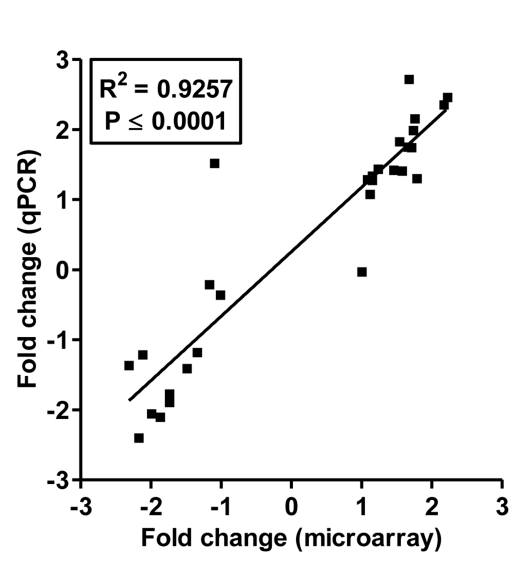
**

**FIGURE B: Validation of microarray results.**

Microarray data were verified by qRT-PCR of RNA from 18 genes of cells incubated with heme or paraquat. RNA from control cells was used as the reference sample. Data were compared with internal Rp49 control and the fold change was obtained using the 2^(-∆∆Ct)^ method. As determined by the significance (p<0.0001) and the Pearson’s correlation coefficient, the qPCR results were consistent with the changes observed in the microarray. The significance was determined using GraphPad Prism (v. 5).

**
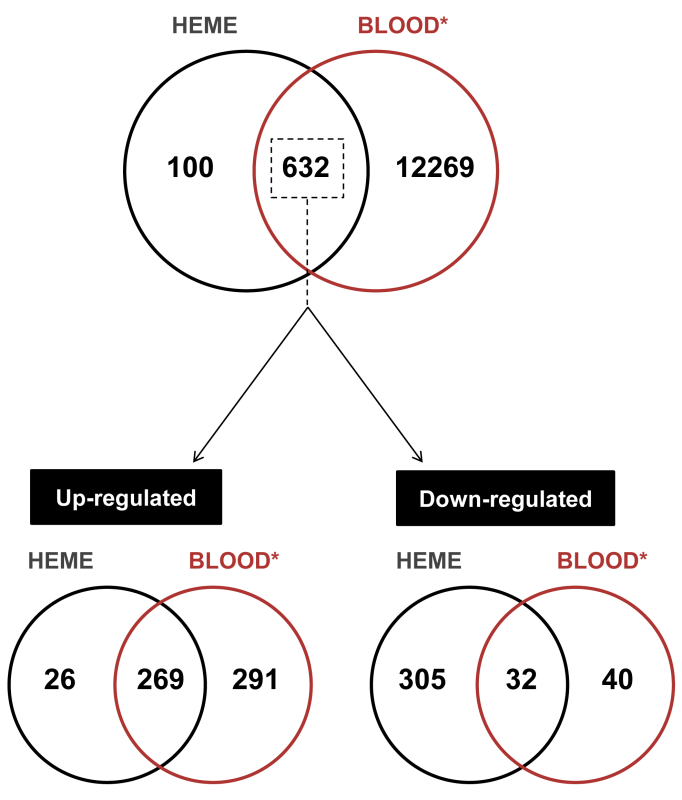
**

**FIGURE C: Analysis of the number of transcripts regulated in both heme-incubated Aag2 cells and blood-fed *Aedes aegypti* females.**

Venn diagram illustrating the numbers of unique and commonly regulated genes in heme-incubated cells and blood-fed females. Among those common transcripts, several were up- or down-regulated in both assays or in only one of them. *Data originally found in: Bonizzoni *et al.* (2011) BMC Genomics 12:82.

**
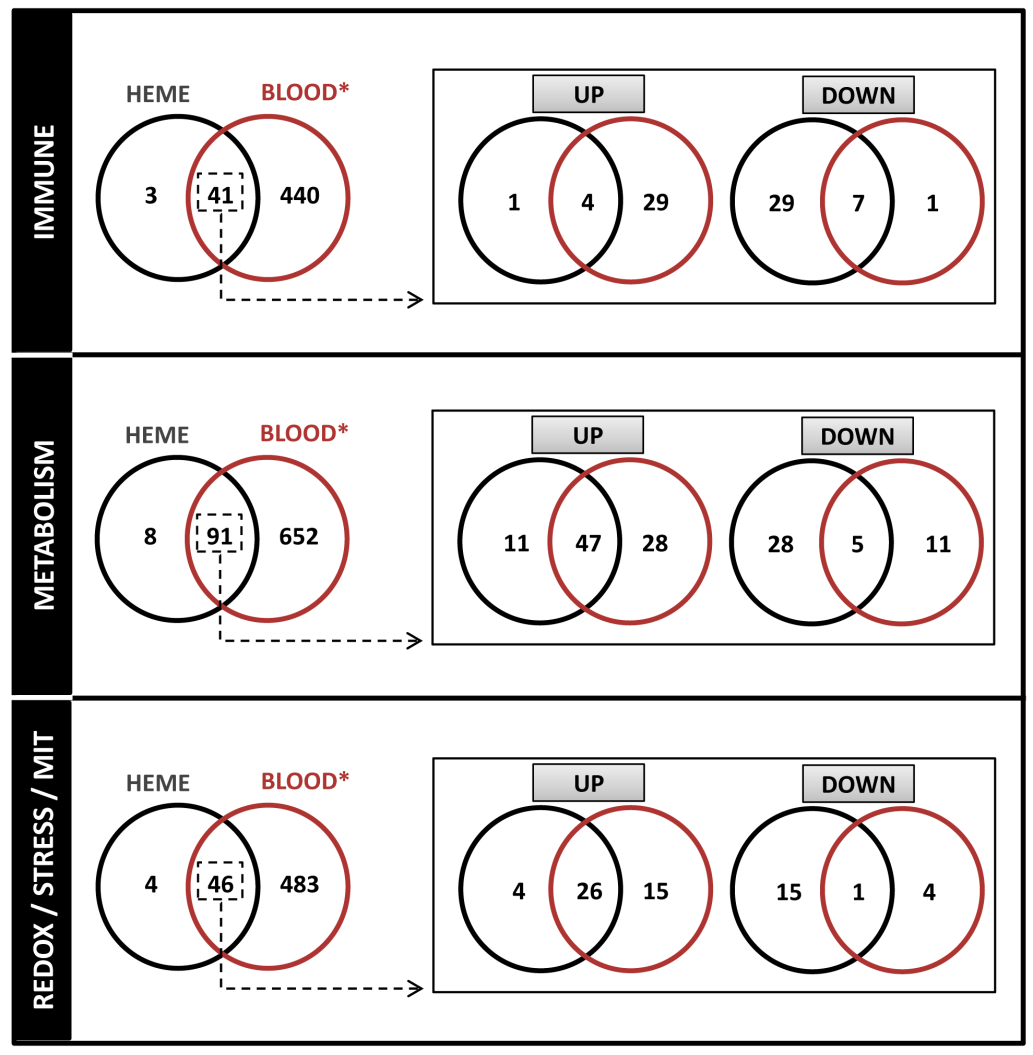
**

**FIGURE D: Functional quantitative analysis of transcripts regulated in both heme-incubated Aag2 cells and blood-fed *Aedes aegypti* females.**

Venn diagrams illustrating the numbers of unique and commonly regulated transcripts in heme-incubated cells and blood-fed females. Top to bottom panels represent data of transcripts functionally associated to immunity, metabolism, or redox, stress and mitochondrion processes, respectively. *Data originally found in: Bonizzoni *et al.* (2011) BMC Genomics 12:82.

**S1 TABLE: Fold change values and functional groups of transcripts that were enriched or depleted in heme- or paraquat-incubated cells.**

Functional group abbreviations: CS, cytoskeletal and structural; CSR, chemosensory reception; DIV, diverse functions; DIG, blood and sugar food digestive; IMM, immunity; MET, metabolism; PROT, proteolysis; RSM, redox, stress and mitochondrion; RTT, replication, transcription, and translation; TRP, transport; UKN, unknown functions. **Excel file*

**S2 TABLE: List of all the transcripts regulated in both heme-incubated Aag2 cells and blood-fed *Aedes aegypti* females.**

The blood-fed transcription data was extracted from then RNA-seq library found in Bonizzoni *et al.* (2011). Transcripts were analyzed according to its pattern of expression – up (+) or down (-) regulated – in both *in vitro* and *in vivo* assays, in comparison to control cells or sugar-fed females, respectively. The main transcripts discussed in the text are highlighted in yellow. Functional group abbreviations are stated above. **Excel file*

**Primers Designed for qRT-PCR Assays**

Alfa-Crystallin F: CAAGGCAACAGACGATAGCA

Alfa-Crystallin R: AGAACACCATCCGACGAGAG

Catalase F: CGGTGGACCAGAACCATGTG

Catalase R: CTTCGGTTTCACCGGACTCG

Cecropin D F: GCTAGGTCAAACCGAAGCAG

Cecropin D R: TCCTACAACAACCGGGAGAG

Cecropin G F: TCACAAAGTTATTTCTCCTGATCG

Cecropin G R: GCTTTAGCCCCAGCTACAAC

CREB F: ggctggtgaaaaatccaaga

CREB R: ctgctcctcaaccactgtca

Defensin A F: GATTCGGCGTTGGTGATAGT

Defensin A R: TTATTCAATTCCGGCAGACG

Dengue (DV2NS5) F: ACAAGTCGAACAACCTGGTCCAT

Dengue (DV2NS5) R: CCGCAGCCATTGGTCTTCTC

DNA-J/hsp40 F: ATCACGTAAGTCCCCGTCCT

DNA-J/hsp40 R: CATTTCATCGGACATCATCG

Ferritin F: GGCAGCAATGACTTCCACTT

Ferritin R : TTTAAGCGTGGCGATCTTCT

GCLC F: CCTCAACGAGAAGGAACACG

GCLC R: GCAGACCACCGTAGGGTTTA

GST F: CTGGAACGCTGCAAATCTCT

GST R: AGGTTCCTCCAGCTTGGAT

Histone H2A F: CAGTTCCCAGTCGGTCGTAT

Histone H2A R: TTTCCTGCCAATTCGAGAAC

HSP 90 F: AAATCAACCCTGACCACTCG

HSP 90 R: AGACCCAGCTTGACCATACG

MAF F: AGACGGAGAAATCGCAGGAA

MAF R: GCAAACTTCCTCAGCGCTTC

NADt F: CATGGCAAGAAGGTTCGTTT

NADt R: ATGATCGAGTTGGGATCGTC

PGRP F: ATTTAACGTCGTGGGAGCAC

PGRP R: TCACTTGGCGATGACCAATA

RP49 F: GCTATGACAAGCTTGCCCCCA

RP49 R: TCATCAGCACCTCCAGCT

TEP F: ATTTTTGACGGCTTTTGTGG

TEP R: TGGATTACTTGCCCCACTTC

Tiorredoxin F: GAACAAGTACGCCGAGAAGG

Tiorredoxina R: ACAGGAAGGTGGGCATACTG

ZIT F: CTCCTCAGCAACGGTTCTTC

ZIT R: CAGGAACTTGATGCCAAACA
